# Structure-guided design of a potent *Clostridioides difficile* toxin A inhibitor

**DOI:** 10.3389/fmicb.2023.1110541

**Published:** 2023-01-26

**Authors:** Greg Hussack, Martin A. Rossotti, Henk van Faassen, Tomohiko Murase, Luiz Eugenio, Joseph D. Schrag, Kenneth K.-S. Ng, Jamshid Tanha

**Affiliations:** ^1^Life Sciences Division, Human Health Therapeutics Research Centre, National Research Council Canada, Ottawa, ON, Canada; ^2^Department of Biological Sciences, University of Calgary, Calgary, AB, Canada; ^3^Life Sciences Division, Human Health Therapeutics Research Centre, National Research Council Canada, Montréal, QC, Canada; ^4^Department of Chemistry and Biochemistry, University of Windsor, Windsor, ON, Canada; ^5^Department of Biochemistry, Microbiology and Immunology, University of Ottawa, Ottawa, ON, Canada

**Keywords:** biparatopic, *Clostridioides difficile*, inhibitor, nanobody, single-domain antibody, toxin, V_H_H

## Abstract

Crystal structures of camelid heavy-chain antibody variable domains (V_H_Hs) bound to fragments of the combined repetitive oligopeptides domain of *Clostridioides difficile* toxin A (TcdA) reveal that the C-terminus of V_H_H A20 was located 30 Å away from the N-terminus of V_H_H A26. Based on this observation, we generated a biparatopic fusion protein with A20 at the N-terminus, followed by a (GS)_6_ linker and A26 at the C-terminus. This A20-A26 fusion protein shows an improvement in binding affinity and a dramatic increase in TcdA neutralization potency (>330-fold [*IC*_50_]; ≥2,700-fold [*IC*_99_]) when compared to the unfused A20 and A26 V_H_Hs. A20-A26 also shows much higher binding affinity and neutralization potency when compared to a series of control antibody constructs that include fusions of two A20 V_H_Hs, fusions of two A26 V_H_Hs, a biparatopic fusion with A26 at the N-terminus and A20 at the C-terminus (A26-A20), and actoxumab. In particular, A20-A26 displays a 310-fold (*IC*_50_) to 29,000-fold (*IC*_99_) higher neutralization potency than A26-A20. Size-exclusion chromatography-multiangle light scattering (SEC-MALS) analyses further reveal that A20-A26 binds to TcdA with 1:1 stoichiometry and simultaneous engagement of both A20 and A26 epitopes as expected based on the biparatopic design inspired by the crystal structures of TcdA bound to A20 and A26. In contrast, the control constructs show varied and heterogeneous binding modes. These results highlight the importance of molecular geometric constraints in generating highly potent antibody-based reagents capable of exploiting the simultaneous binding of more than one paratope to an antigen.

## Introduction

*Clostridioides difficile* is a spore-forming Gram-positive bacterium capable of infecting humans and causing symptoms ranging from mild diarrhea to pseudomembranous colitis (Hussack and Tanha, 2016; Kordus et al., 2022). *C. difficile* infection is one of the most prevalent hospital-acquired bacterial infections, costing health-care systems ~$5 billion per year and claiming up to 30,000 lives annually in the United States alone (Fu et al., 2021). The healthy balance of microbiota normally present in the gastrointestinal tract of individuals is thought to suppress *C. difficile* infection (Vasilescu et al., 2021). Changes to this balance, often *via* the introduction of broad-spectrum antibiotics, can increase individuals’ susceptibility to *C. difficile* colonization and infection. *C. difficile* remains an Urgent Threat pathogen according to the Centers for Disease Control and Prevention, despite numerous advances in new therapeutic agents to treat *C. difficile* infection.^1^

*C. difficile* infection is currently treated with antibiotics that include metronidazole, vancomycin and fidaxomicin; however, significant incidences of disease relapse have made the search for alternative treatments, including vaccines (Henderson et al., 2017), fecal transplantation (Shogbesan et al., 2018) and antibody-based immunotherapy (Hussack and Tanha, 2016), a top public health priority. The primary virulence factors of *C. difficile* are two high molecular weight toxins (Kordus et al., 2022), toxin A (TcdA) and toxin B (TcdB), which are largely responsible for the physical damage in the colon of infected individuals and are the foremost targets of vaccine- and immunotherapy-based approaches (Hussack and Tanha, 2016; Henderson et al., 2017). Recently, a monoclonal antibody (mAb) targeting TcdB was shown to significantly reduce *C. difficile* relapse and was approved by the Food and Drug Administration. The trial data showed that reduced incidences of relapse were driven solely by the anti-TcdB mAb (bezlotoxumab) and not enhanced by the inclusion of an anti-TcdA mAb (actoxumab) in combination with bezlotoxumab (Wilcox et al., 2017). Interestingly, neither the TcdB antibody alone nor both mAbs in combination had a significant impact on the duration or severity of *C. difficile* infection (Wilcox et al., 2017), possibly due to the relatively poor efficacy of actoxumab. Several studies have illustrated that circulating anti-TcdA and TcdB antibodies in human patients correlate with reduced recurrence (Kyne et al., 2000, 2001; Leav et al., 2010), suggesting the development of TcdA inhibitors with considerably greater potency than actoxumab are warranted for use in combination with bezlotoxumab or other TcdB inhibitors. Efforts toward developing anti-toxin Ab-based therapeutics, including therapeutic approaches targeting both TcdA and TcdB, have been reviewed earlier (Hussack and Tanha, 2016).

Toward the goal of developing more effective therapeutics targeting TcdA, we previously isolated a panel of camelid single-domain antibodies (V_H_Hs or nanobodies) targeting *C. difficile* TcdA, including several V_H_Hs with the ability to neutralize TcdA cytotoxicity (Hussack et al., 2011). To better understand the determinants of molecular recognition and neutralization activity, we solved the crystal structures of two of these V_H_Hs targeting non-overlapping TcdA epitopes, namely A20.1 (A20) and A26.8 (A26), in complex with fragments from the combined repetitive oligopeptides (CROPs) domain of TcdA (Murase et al., 2014). These high-resolution crystal structures identified the specific locations of unique epitopes targeted by these antibodies and explained the synergistic TcdA neutralizing effects observed with these V_H_Hs (Hussack et al., 2011). While monomeric V_H_Hs and combinations of monomeric V_H_Hs showed modest TcdA inhibition *in vitro*, we hypothesized that assembling the V_H_Hs as multimers and biparatopic dimers may improve TcdA neutralizing potency. Combining V_H_Hs in tandem, targeting distinct and/or common epitopes, has been successfully demonstrated for a number of anti-toxin V_H_H systems, including: botulinum toxins (Mukherjee et al., 2012; Huang et al., 2017; Lam et al., 2020; Tremblay et al., 2020), anthrax toxin (Moayeri et al., 2015; Vrentas et al., 2016), ricin (Vance et al., 2013; Herrera et al., 2015, 2016), scorpion toxins (Hmila et al., 2008, 2010), Shiga toxins (Tremblay et al., 2013; Mejías et al., 2016), tetanus toxin (Rossotti et al., 2015), α-neurotoxin (Wade et al., 2022) and *C. difficile* TcdA and TcdB (Yang et al., 2014; Schmidt et al., 2016). In many of these examples, V_H_H orientations were largely chosen at random, and the spacer lengths separating the two (or more) V_H_H domains were typically not extensively explored.

**Figure 1:**
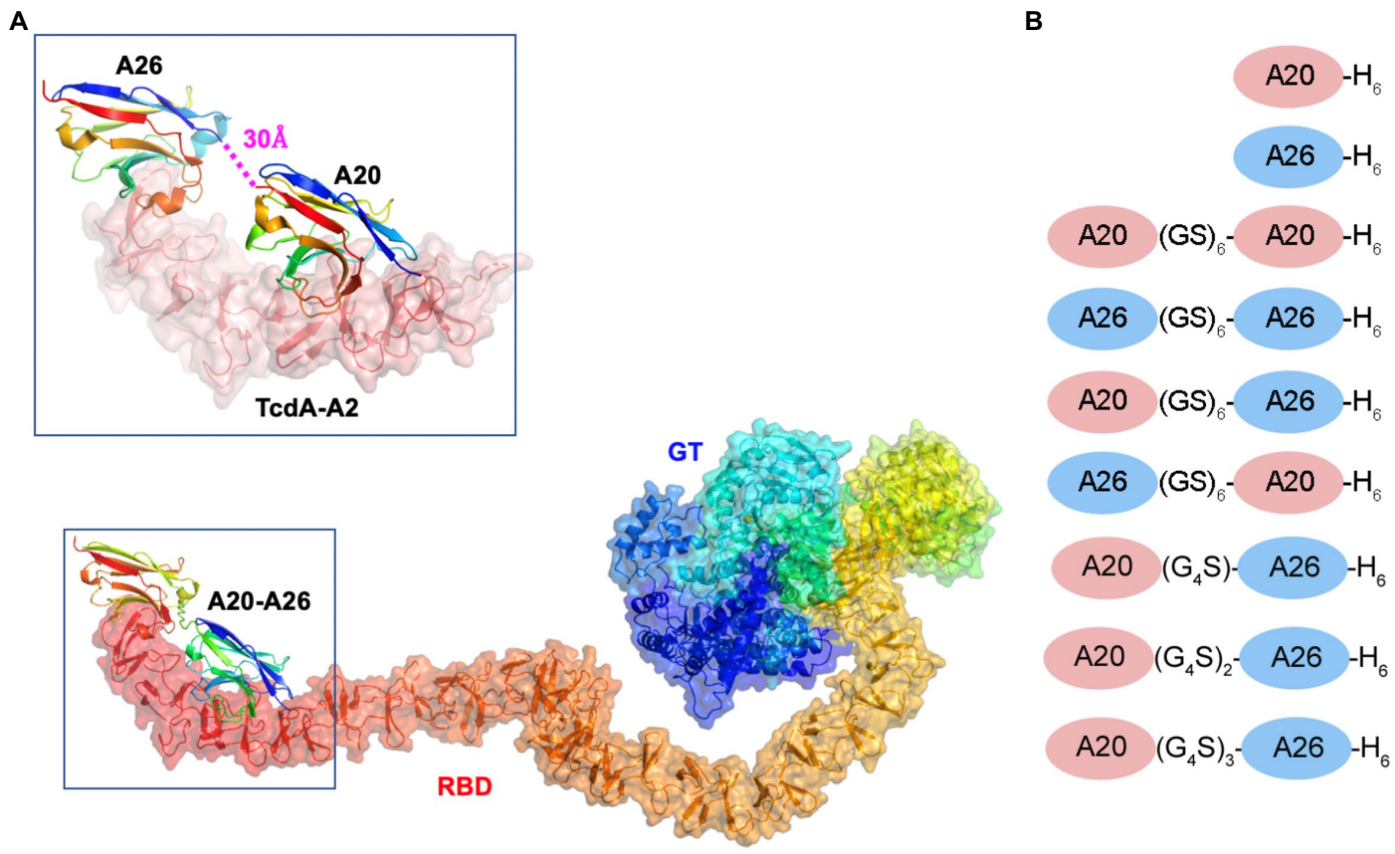
Model of the A20-A26 fusion protein bound to the CROPs domain of *Clostridioides difficile* TcdA. **(A)** The structure of full-length *C. difficile* TcdA was generated by superimposing the crystal structure of the N-terminal 1832 amino acid residues of TcdA (4R04) (Chumbler et al., 2016) onto the crystal structure of TcdB (6OQ5) (Chen et al., 2019). To model the CROP domain missing from the TcdA structure, we made the assumption that the orientation of the CROP domain relative to the N-terminal domains is similar in the two toxins. By superimposing the N-terminal short repeat of a model of the TcdA CROP domain (Ho et al., 2005; Pruitt et al., 2010; Pruitt and Lacy, 2012) onto the N-terminal short repeat in the CROP domain of TcdB (6OQ5), a model of full-length TcdA was generated. The resulting model shows similar features to the low resolution cryo-EM envelopes of full-length TcdA (Pruitt et al., 2010; Pruitt and Lacy, 2012). The structure of the complex formed between A20-A26 and TcdA was generated by modeling the structure of a (GS)_6_ linker in an extended antiparallel β-strand conformation using PyMOL and positioning this linker between the C-terminus of the A20 V_H_H and the N-terminus of the A26 V_H_H observed in the crystal structure of the complex formed between A20, A26 and the TcdA-A2 fragment (4NC1) (Murase et al., 2014). TcdA is shown in cartoon ribbon and semi-transparent surface representations. A20, A26 and the A20-A26 V_H_H domains are drawn in cartoon representation. Each polypeptide chain is colored according to the colors of the rainbow, starting from blue at the N-terminus to red at the C-terminus. RBD, receptor binding domain; GT, glucosyl transferase. **(B)** Schematic representation of the V_H_H constructs generated in this work.

The crystal structure of A20 and A26 bound to the C-terminal 255 residues of TcdA (amino acid residues 2,556–2,710 of TcdA10463; referred to as TcdA-A2 fragment; PDB 4NC1) revealed that the C-terminus of A20 was located 30 Å away from the N-terminus of A26 (Murase et al., 2014; Figure 1A). Molecular modeling led us to hypothesize that connecting A20 to A26 by a 12 residue (Gly-Ser)_6_ linker would generate a biparatopic fusion protein in which the two tethered V_H_H domains could simultaneously bind to a single molecule of TcdA, potentially generating a TcdA inhibitor with substantially higher binding affinity and inhibitory potency. To test this hypothesis, we have produced this biparatopic fusion protein and used *in vitro* binding and cytotoxicity inhibition measurements to confirm its high potency and specificity, capable of neutralizing TcdA at picomolar concentrations. The much weaker neutralization activities observed for a series of control constructs highlight the advantages of a structure-based approach in designing highly specific neutralization reagents and define some of the geometric constraints that are most critical for successfully combining V_H_Hs into biparatopic reagents. This study provides a dramatic example of how high-resolution crystal structures can be used to tailor the design of a multivalent toxin-inhibiting protein to match the molecular geometry of a toxin to generate novel and potent therapeutics.

## Materials and methods

### Structure-guided design of V_H_H fusion proteins

The ternary complex of the TcdA-A2 fragment bound to A20 and A26 (PDB 4NC1) indicated that the N-terminus of A26 was located 30 Å away from the C-terminus of A20. Models of linkers containing a repeating Gly-Ser dipeptide motif with backbone geometry similar to that seen in antiparallel β-strands were generated using PyMOL (Version 2.0, Schrödinger LLC). A linker with six (GS) dipeptides appeared to be sufficient to bridge the gap observed in the 4NC1 crystal structure, but the presence of disordered residues at the N- and C-termini of the V_H_H proteins introduced a modest degree of uncertainty into the design. As a result, a series of constructs with a small range of linker lengths were generated.

### Antibody expression and purification

V_H_Hs A20, A26, and B39 [anti-TcdB control V_H_H; (Hussack et al., 2018)] were expressed in *Escherichia coli* TG1 cells as described previously (Hussack et al., 2011). The optimized DNA sequences encoding A20-A20, A26-A26, A26-A20, A20-A26 (Hussack et al., 2011), and B39-B39 (Hussack et al., 2018) dimers were synthesized and subcloned into pTT5 (Durocher et al., 2002) expression plasmid (Thermo Fisher, Ottawa, Canada). The two monomer units in each dimer are separated by a (Gly-Ser)_6_ linker sequence. Three additional A20-A26 constructs with Gly_4_Ser, (Gly_4_Ser)_2_, and (Gly_4_Ser)_3_ amino acid linkers, hereafter referred to as A20-A26 (G_4_S), A20-A26 (G_4_S)_2_, and A20-A26 (G_4_S)_3_, were also subcloned into pTT5. All V_H_H dimers, the anti-TcdA IgG1 reference antibody actoxumab (CDA1) and the anti-TcdB IgG1 reference antibody bezlotoxumab (MDX1388) were expressed in HEK293-6E cells as described (Hussack et al., 2018). Five days after the transfection, supernatants were harvested by centrifugation at 3,000 × *g* for 10 min and filtered through 0.2-μm filter (Millipore, Etobicoke, Canada), dialyzed against phosphate-buffered saline (PBS) overnight at 4°C, and then the His_6_-tagged dimers were purified by immobilized metal-ion affinity chromatography using a HisTrap column (Cytiva Life Sciences, Mississauga, Canada) and an AKTÄ™ FPLC (Cytiva Life Sciences). CDA1 and MDX1388 were purified by protein A affinity chromatography. The eluted proteins were buffer exchanged into PBS using Amicon devices, sterilized through 0.2-μm filtration and stored at −80°C. Antibody concentrations were determined from their respective molar extinction coefficients and A_280_ absorbance, measured on a NanoDrop 3300 fluorospectrometer (Thermo Fisher). Protein purity was assessed by reducing and non-reducing sodium dodecyl sulfate–polyacrylamide gel electrophoresis (SDS-PAGE).

### Size-exclusion chromatography

Purified proteins (400 μg in PBS) were loaded onto a Superdex™ 75 Increase 10/30 GL (Cytiva Life Sciences) column, at a flow rate of 0.5 mL/min, controlled by an AKTÄ™ FPLC (Cytiva Life Sciences). To determine the size of each construct, a standard curve was generated using the Bio-Rad™ Size-Exclusion Chromatography Standard (BioRad, Hercules, CA) and apparent molecular masses (*M*_app_s) of antibodies were calculated by interpolation using their elution volumes (*V*_e_s). Size-exclusion chromatography (SEC) chromatograms were normalized as described (Kim et al., 2012).

### Enzyme-linked immunosorbent assay

Nunc-immuno microtitre plate wells with Maxisorp surface (Thermo Fisher) were coated in triplicates with 30 ng/well of full-length TcdA (List Biological Laboratories, Inc., Campbell, CA) in 0.05 M carbonate–bicarbonate buffer pH 9.6, and incubated overnight at 4°C. Wells were blocked with 1% (w/v) bovine serum albumin in PBS for 1 h at room temperature, washed with PBS/0.05% (v/v) Tween 20 and then incubated for 1 h with decreasing concentrations of His_6_-tagged V_H_H constructs in PBS containing 0.2% bovine serum albumin and 0.05% Tween 20 (PBS-TB). Following washing, the binding to TcdA was probed by incubating the wells for 1 h at room temperature with 0.2 ng/mL polyclonal rabbit anti-His_6_ antibody conjugated to horseradish peroxidase (Bethyl Laboratories, Montgomery, TX) in PBS-TB. Wells were washed and incubated at room temperature for 15 min with 100 μL TMB peroxidase substrate solution (KPL, Gaithersburg, MD). Then, 50 μL of 1 M H_2_SO_4_ was added to stop enzymatic reactions and absorbance was read at 450 nm on a Multiskan Enzyme-linked immunosorbent assay (ELISA) plate reader (Thermo Fisher). Data analysis was performed using Prism software version 8.3 (GraphPad Software, Inc., La Jolla, CA).

### Surface plasmon resonance analysis

All surface plasmon resonance (SPR) experiments were performed on a Biacore 3000 instrument (Cytiva Life Sciences) at 37°C in HBS-EP running buffer (10 mM HEPES, pH 7.4, 150 mM NaCl, 3 mM EDTA, 0.005% (v/v) surfactant P20). Recombinant TcdA-A2 fragment (Greco et al., 2006), encompassing the C-terminal CROPs domain of TcdA (aa 2,556–2,710) from *C. difficile* reference strain 10463, was amine coupled to a CM5 sensor chip at pH 4.5 in acetate buffer using standard methods recommended by the manufacturer (Cytiva Life Sciences) to create a high-density toxin A surface with ~8,500 resonance units immobilized. An ethanolamine-blocked flow cell served as a reference surface. The flow rate for all experiments was 40 μL/min and SEC-purified antibodies were injected for 120 s at a single concentration that varied depending on the antibody (CDA1: 50 nM; A26 and B39: 10 nM; A20: 5 nM; A20-A20, A26-A26, A20-A26 and A26-A20: 1 nM). Antibody dissociation was followed for 300 s for the monomeric antibodies (A20, A26, and B39) and 3,600 s for the bivalent antibodies (A20-A20, A26-A26, A20-A26, A26-A20 and CDA1). Complete regeneration of the TcdA-A2 surface was achieved with a 6 s pulse of 10 mM glycine, pH 2.0, for all antibodies except CDA1 (12 s pulse of 5 mM NaOH), all at a flow rate of 100 μL/min. Off-rates (*k*_d_s, s^−1^) were determined by fitting the dissociation phase (1,500–3,500 s for bivalent antibodies, 150–300 s for A20, 130–200 s for A26) of each sensorgram to a separate *k*_d_ 1:1 binding model using the BIAevaluation v4.1 software (Cytiva Life Sciences). With one exception (A20-A26), all antibodies achieved the minimum 5% dissociation required to accurately report an off-rate (Katsamba et al., 2006).

### *In vitro* TcdA neutralization assay

Vero cells (CCL-81) were obtained from ATCC (Manassas, VA) and cultured according to ATCC’s instructions in 96 well microtiter plates (Nunc) at 2 × 10^4^ cells/well in MEM media (Gibco) supplemented with 10% (v/v) heat-inactivated fetal bovine serum (Gibco) at 37°C in 5% CO_2_. The neutralization activity of antibodies was determined by co-incubation with 80 ng/mL (260 pM) of full-length TcdA, with antibody and toxin added simultaneously to the cells. After 72 h of incubation, the cell viability was quantified with the Cell Proliferation Assay Reagent WST-1 (Roche Diagnostics, Laval, Canada) according to the manufacturer’s instructions. Briefly, the media was replaced with 100 μL of MEM (without fetal bovine serum) containing 10% of WST-1, incubated for 40 min at 37°C in 5% CO_2_ and finally the absorbance read at 450 nm. The neutralizing activity was calculated as % inhibition:


$$\%\;\mathrm{Inhibition}=\frac{A450_{\mathrm{test}}-A450_{\mathrm{low}}}{A450_{\mathrm{high}}-A450_{\mathrm{low}}}\times 100$$

A450_test_ is the absorbance of cells incubated with TcdA and varying concentrations of antibodies;

A450_low_ is the absorbance of cells incubated only with TcdA (0% inhibition); and

A450_high_ is the absorbance of cells incubated only with media (100% inhibition).

### UPLC-SEC-multiangle light scattering analysis

Monomeric and dimeric V_H_Hs were mixed with TcdA-A2 at 1:1 molar ratios in PBS at final concentrations of 1.91 mg/mL and incubated at 4°C overnight. Control experiments which included monomeric V_H_Hs alone (0.91 mg/mL), dimeric V_H_Hs alone (0.91 mg/mL) and TcdA-A2 alone (1 mg/mL) were performed under the same conditions as the V_H_H-TcdA-A2 mixtures. UPLC-SEC analysis of protein samples was performed on a BEH200 SEC column (4.6 × 150 mm, 1.7 μm particle column, Waters, Milford, MA) using a Waters H-Class Acquity UPLC system equipped with a diode array detector, a Wyatt MiniDawn multiangle light scattering (MALS) detector, and a Wyatt Optilab T-rEX refractive index detector (Wyatt Technology, Santa Barbara, CA). The mobile phase was PBS (HyClone SH30028.02, Cytiva Life Sciences) containing 0.02% (v/v) polysorbate 20 at a flow rate of 0.4 mL/min. The column temperature was 30°C. Molar masses were determined using Astra software version 6.1.7.17 (Wyatt Technology) using either absorbance at 280 nm (A_280_) or refractive index (RI) as the concentration measure. Similar results were obtained using either A_280_ or RI, but band broadening reduced resolution in the RI signal so masses determined using the A_280_ signal are reported.

## Results

### Structure-guided design of V_H_H fusion proteins

We previously solved the *X*-ray structures of two *C. difficile* TcdA-binding V_H_Hs, A20 (*K*_D_ = 2 nM) and A26 (*K*_D_ = 12 nM), in complex with fragments from the CROPs domain (formerly referred to as the RBD, receptor binding domain) of TcdA (Hussack et al., 2011; Murase et al., 2014). Because in the ternary complex of the TcdA-A2 fragment bound to a single molecule of A20 and a single molecule of A26 (PDB 4NC1) the N-terminus of A26 was observed to be located 30 Å away from the C-terminus of A20, we hypothesized that a short linker peptide could be used to fuse the two V_H_H domains together in a recombinant fusion protein (Figure 1). Models of linkers containing a repeating Gly-Ser dipeptide motif were generated with backbone geometry similar to that seen in antiparallel β-strands. A linker with six Gly-Ser dipeptides (GS)_6_ appeared to be sufficient to bridge the gap observed in the 4NC1 crystal structure, but the presence of disordered residues at the N- and C-termini of the V_H_H proteins indicate that the ideal lengths and geometric details of linkers is challenging to model precisely. As a result, in addition to A20-A26 with a (GS)_6_ linker, a series of constructs with a range of linker lengths were generated (Figure 1B). These included A20-A26 (G_4_S), A20-A26 (G_4_S)_2_, and A20-A26 (G_4_S)_3_, which possessed linkers of shorter [(G_4_S)], similar [(G_4_S)_2_] and longer [(G_4_S)_3_] distances than the (GS)_6_ construct. The crystal structures also clearly indicated that the polarity of the arrangement of V_H_H domains (i.e., whether the A20 V_H_H domain or the A26 V_H_H domain is N-terminal to the other V_H_H domain in the fusion protein) would likely be critical, as the reverse orientation of V_H_H domains would not be expected to allow for the simultaneous binding of both V_H_H domains to the same molecule of TcdA. To test this prediction, the A26-A20 fusion protein was generated with the reverse polarity (i.e., A26 at the N-terminus and A20 at the C-terminus). To evaluate whether the tethering of two V_H_H domains generates a non-specific multivalency effect, two other fusion proteins containing two A20 V_H_H domains (A20-A20) and two A26 V_H_H domains (A26-A26), each tethered by the (GS)_6_ linker, were also generated.

### Expression, purification and biophysical characterization of V_H_H fusion proteins

**Figure 2:**
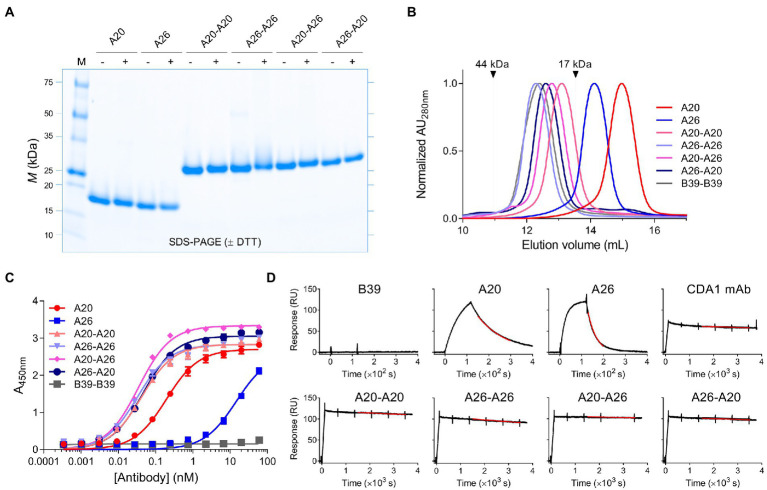
Biophysical characterization of anti-TcdA V_H_H constructs. **(A)** Representative SDS-PAGE analysis of V_H_H monomers and dimers is shown under reducing (+DTT [dithiothreitol]) and non-reducing (−DTT) conditions. M, protein molecular mass standards AU, absorbance unit. **(B)** SEC analysis of the antibodies with arrows indicating the elution positions of ovalbumin (44 kDa) and myoglobulin (17 kDa) protein standards. **(C)** Titration of the antibodies against full-length TcdA by ELISA. B39-B39 (a dimer of the anti-TcdB V_H_H B39 with a (GS)_6_ linker) is used as a negative control. Error bars indicate standard deviation (SD) of three technical replicates. **(D)** Representative SPR sensorgrams comparing off-rate of V_H_Hs, V_H_H dimers and CDA1 mAb binding to immobilized TcdA-A2 fragment at 37°C with 5min or 60min dissociations. Black lines represent raw data points; red lines are fits to a separate *k*_d_ 1:1 binding model. RU, resonance unit.

While monomeric V_H_Hs were expressed in the periplasm of *E. coli*, the dimeric V_H_Hs were expressed in mammalian HEK293-6E cells to obtain higher expression yields. Nonetheless, in contrast to A20-A26 (G_4_S) and A20-A26 (G_4_S)_2_, which could be expressed in high yields similar to the dimers with the (GS)_6_ linker (Table 1), the A20-A26 (G_4_S)_3_ construct was surprisingly only expressed at very low yields despite repeated attempts, excluding its full analysis in the current study. The proteins were purified by immobilized metal-ion affinity chromatography and ran as single bands of the expected molecular masses on SDS-PAGE under reducing and non-reducing conditions (Figure 2A). Control proteins (V_H_H B39, V_H_H-V_H_H B39-B39, mAbs CDA1 and MDX1388) were expressed in *E. coli* or HEK293-6E cells and purified by immobilized metal-ion affinity chromatography or protein A affinity chromatography (data not shown). Purified yields of A20 and A26 were in the range of 15–20 mg/L, and yields of the four dimeric V_H_Hs ranged from 80–114 mg/L (Table 1). The SEC profiles of all V_H_Hs and V_H_H dimers produced single, monodispersed peaks devoid of aggregates (100% “monomeric”) with *V*_e_s consistent for a V_H_H monomer to dimer transition and relative to protein standards (Figure 2B; Table 1).

**Table 1:** Biophysical properties of anti-TcdA V_H_H constructs.

Antibody	Linker	*M*_r_ (Da)	Yield (mg/L)	Mon. (%)^c^	*EC*_50_ (pM)^d^	*k*_d_ (s^−1^)^e^	Dissociation (%)^f^	*T*_1/2_ (h)^g^
A20	na	15,670	19^a^	100	194 ± 13	(7.5 ± 0.1) × 10^−3^	88.4 ± 0.3	0.03
A26	na	16,016	15^a^	100	14,620 ± 2,320	(2.5 ± 0.1) × 10^−2^	98.9 ± 0.2	0.01
A20-A20	[GS]_6_	28,679	80^b^	100	42 ± 3	(1.8 ± 0.2) × 10^−5^	7.7 ± 1.1	10.6
A26-A26	[GS]_6_	29,371	110^b^	100	32 ± 3	(3.6 ± 0.4) × 10^−5^	13.5 ± 0.7	5.4
A20-A26	[GS]_6_	29,025	114^b^	100	34 ± 2	(6.9 ± 1.5) × 10^−6^	3.9 ± 0.5	28.1
A26-A20	[GS]_6_	29,025	112^b^	100	47 ± 2	(1.7 ± 0.2) × 10^−5^	7.5 ± 1.1	11.3
CDA1 IgG	na	145,580	80	nd	nd	(2.2 ± 0.2) × 10^−5^	18.0 ± 0.6	8.8

na, not applicable; nd, not determined. ^a^Protein purification yield from 1 L *E. coli* cultures. ^b^Protein purification yield extrapolated from 100 mL HEK293-6E cultures. ^c^Percent monomer peak area determined from size exclusion chromatograms. ^d^Determined by ELISA. ^e^Determined by SPR at 37°C with a 5 min (monomers) or 60 min (dimers, mAb) dissociation. Values are mean ± SD from three technical replicates. ^f^Percent dissociation of SPR response after 5 min (monomers) or 60 min (dimers, mAb). Values are mean ± SD from three technical replicates. ^g^SPR-calculated half-life (*T*_1/2_ = 0.693/*k*_d_).

Binding of V_H_Hs and V_H_H-V_H_Hs to immobilized TcdA was demonstrated by ELISA (Figure 2C; Table 1). The four dimeric constructs, A20-A20, A26-A26, A20-A26 and A26-A20, showed the strongest binding to TcdA with similar apparent *EC*_50_s of 32–47 pM. In contrast, monomeric A20 and A26 demonstrated significantly lower TcdA binding, with *EC*_50_s of 194 pM (A20) and 14.6 nM (A26). The off-rates (*k*_d_s, s^−1^) of all constructs were determined by SPR by flowing V_H_Hs, V_H_H-V_H_Hs or control CDA1 mAb over amine coupled TcdA for long dissociation times at 37°C (Figure 2D; Table 1). Consistent with the ELISA results, A20 and A26 dissociated rapidly with *k*_d_s of 2.5 × 10^−2^ s^−1^ and 7.5 × 10^−3^ s^−1^, respectively. All dimeric constructs clearly showed avid binding and demonstrated very slow dissociation rates as expected; however, subtle differences were evident. Of the dimeric proteins, A26-A26 dissociated the fastest followed by A20-A20 and A26-A20, which had similar *k*_d_s to the CDA1 IgG benchmark. The dissociation of A20-A26 was very slow (6.9 × 10^−6^ s^−1^), which is at the instrument limit of detection, achieving only 4% dissociation after an hour and corresponding to an estimated half-life of 28.1 h (Table 1).

### *In vitro* TcdA neutralization

**Figure 3:**
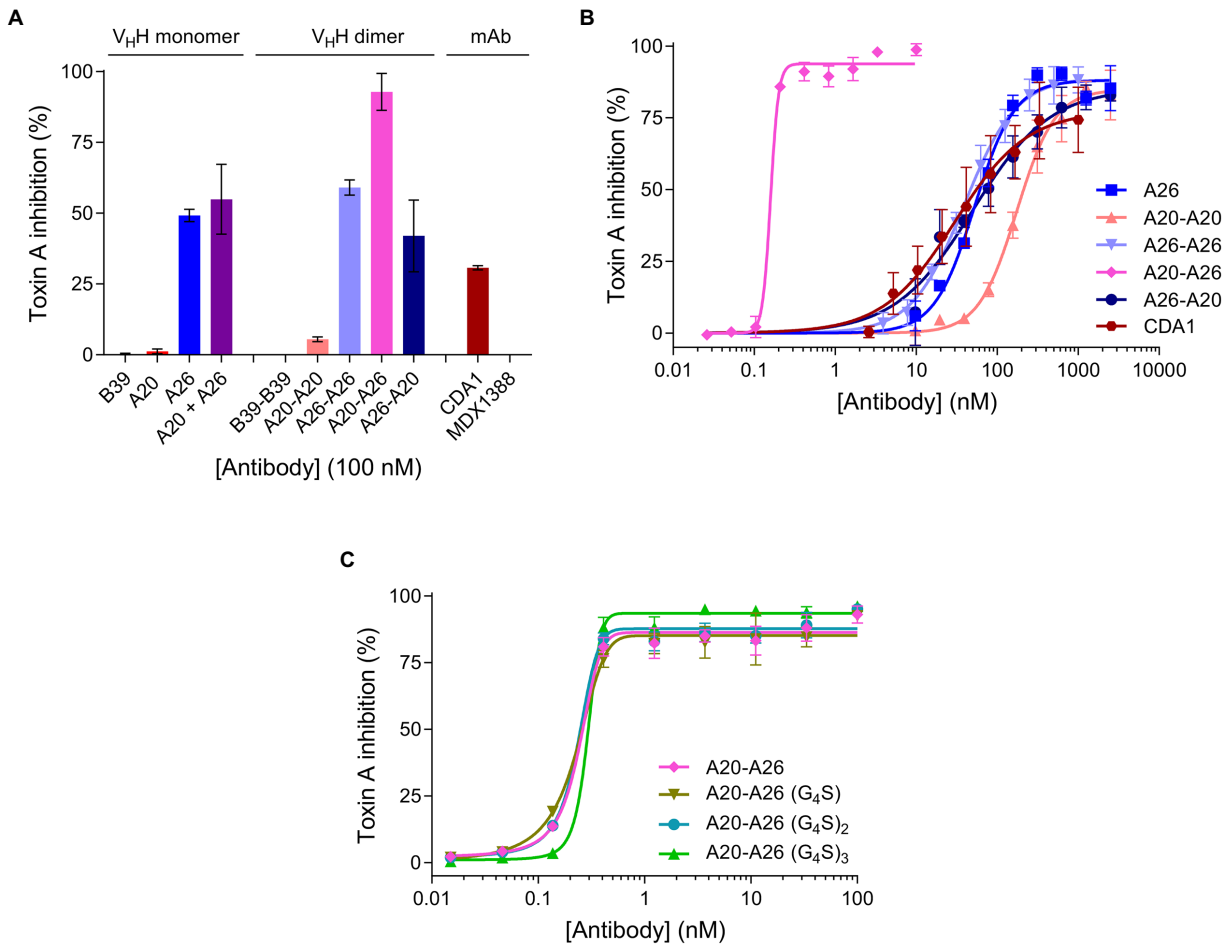
*In vitro* neutralization of TcdA cytotoxicity. **(A)** The panel of antibodies at 100 nM were incubated with TcdA for 72 h and the percentage of live Vero cells were quantified by the colorimetric proliferation reagent WST-1. MDX1388 (anti-TcdB mAb) and B39-B39 (a dimer of the B39 V_H_H with a (GS)_6_ linker) were negative control antibodies. **(B)** The most potent antibodies were further titrated to evaluate their performance based on potency (*IC*_50_ and *IC*_99_) and efficacy (maximum inhibition) values. **(C)** Comparison of neutralizing potency of the structure-guided A20-A26 construct containing a 12-amino acid linker, to A20-A26 constructs containing 5, 10, and 15 amino acid linkers. In all assays the TcdA concentration was 260 pM and antibody concentrations as described. Error bars indicate standard deviation (SD) of three technical replicates.

We compared the TcdA neutralizing potency of the various V_H_H constructs and controls in a 72 h TcdA inhibition assay using Vero cells. Initially, toxin neutralization was performed on monomers, dimers and the CDA1 benchmark mAb at a concentration of 100 nM (Figure 3A). The A20-A26 construct was a superior neutralizer in comparison to other monomers (A20, A26, and A20 + A26), dimers (A20-A20, A26-A26, and A26-A20) and CDA1, reaching nearly 100% TcdA neutralization. Moreover, while A26 demonstrated significant TcdA inhibition, A20 did not show any despite its higher affinity for TcdA, underlining the critical role epitope location plays in TcdA neutralization. Next, we performed neutralization experiments with various antibody concentrations (Figure 3B) to determine *IC*_50_, *IC*_99_ and maximum toxin inhibition values (Table 2). Antibody titration curves demonstrated a dramatic shift in neutralizing potency of A20-A26 relative to the other mono- and biparatopic constructs tested. The *IC*_50_ of A20-A26 (0.16 nM) was far superior to A26-A20 (49.7 nM), A20-A20 (174.5 nM), A26-A26 (39 nM), and CDA1 (30 nM). The *IC*_99_ of A20-A26 (0.27 nM) was even more dramatic in relation to comparators A26-A20 (7,866 nM), A20-A20 (2,123 nM), A26-A26 (1,189 nM) and CDA1 (3,342 nM). In particular, relative to the reverse orientation control A26-A20 and CDA1, A20-A26 outperformed these antibodies by 29,000- and 12,500-fold, respectively. Antibody efficacy, reported as the maximum level of TcdA inhibition achieved, was also superior for the A20-A26 construct reaching 94% compared to A26-A20 (85%), A20-A20 (85%), A26-A26 (89%) and CDA1 (78%).

**Table 2:** TcdA neutralizing potency and efficacy of V_H_H constructs.

Antibody	Linker	TcdA inhibition		
		*IC*_50_ (nM)	*IC*_99_ (nM)^a^	Efficacy (%)^b^
A20	na	–	–	–
A26	na	52.6 ± 3.2	719	88.1 ± 1.7
A20-A20	[GS]_6_	174.5 ± 12.5	2,123	84.9 ± 2.4
A26-A26	[GS]_6_	39.0 ± 2.9	1,189	89.1 ± 2.1
A20-A26	[GS]_6_	0.16 ± 0.01	0.27	93.8 ± 1.0
A26-A20	[GS]_6_	49.7 ± 9.1	7,866	85.3 ± 4.2
CDA1 IgG	na	30.0 ± 8.1	3,342	77.5 ± 6.2
A20-A26 (G_4_S)	[G_4_S]	0.22 ± 0.02	0.54	85.1 ± 1.2
A20-A26 (G_4_S)_2_	[G_4_S]_2_	0.23 ± 0.02	0.42	87.7 ± 1.1
A20-A26 (G_4_S)_3_	[G_4_S]_3_	0.29 ± 0.02	0.46	93.5 ± 0.8

Vero cell cytotoxicity assays measuring mean ± SD TcdA inhibition after 72 h, from three technical replicates, shown in Figures 3B,C. na, not applicable; −, lack of neutralization activity (at 100 nM V_H_H concentration). ^a^Calculated from mean *IC*_50_ value and Hill slope. ^b^Maximum level of TcdA inhibition.

Given the potency of A20-A26, three additional constructs were designed with varying linker lengths of (G_4_S), (G_4_S)_2_, and (G_4_S)_3_ separating the two V_H_Hs. Neutralization experiments comparing the original A20-A26 construct containing the (GS)_6_ linker with the various G_4_S linkers revealed essentially identical neutralizing potency (Figure 3C). This is reflected in similar *IC*_50_ (0.16 nM, 0.22 nM, 0.23 nM, and 0.29 nM) and *IC*_99_ (0.27 nM, 0.54 nM, 0.42 nM, and 0.46 nM) values for A20-A26, A20-A26 (G_4_S), A20-A26 (G_4_S)_2_, and A20-A26 (G_4_S)_3_, respectively (Table 2). The antibody efficacy of A20-A26 (94%) slightly exceeded constructs with shorter linkers A20-A26 (G_4_S) (85%) and A20-A26 (G_4_S)_2_ (88%), and was essentially the same as the construct with a longer linker A20-A26 (G_4_S)_3_ (94%) (Table 2).

### UPLC-SEC-MALS analysis of V_H_H-TcdA complexes

**Figure 4:**
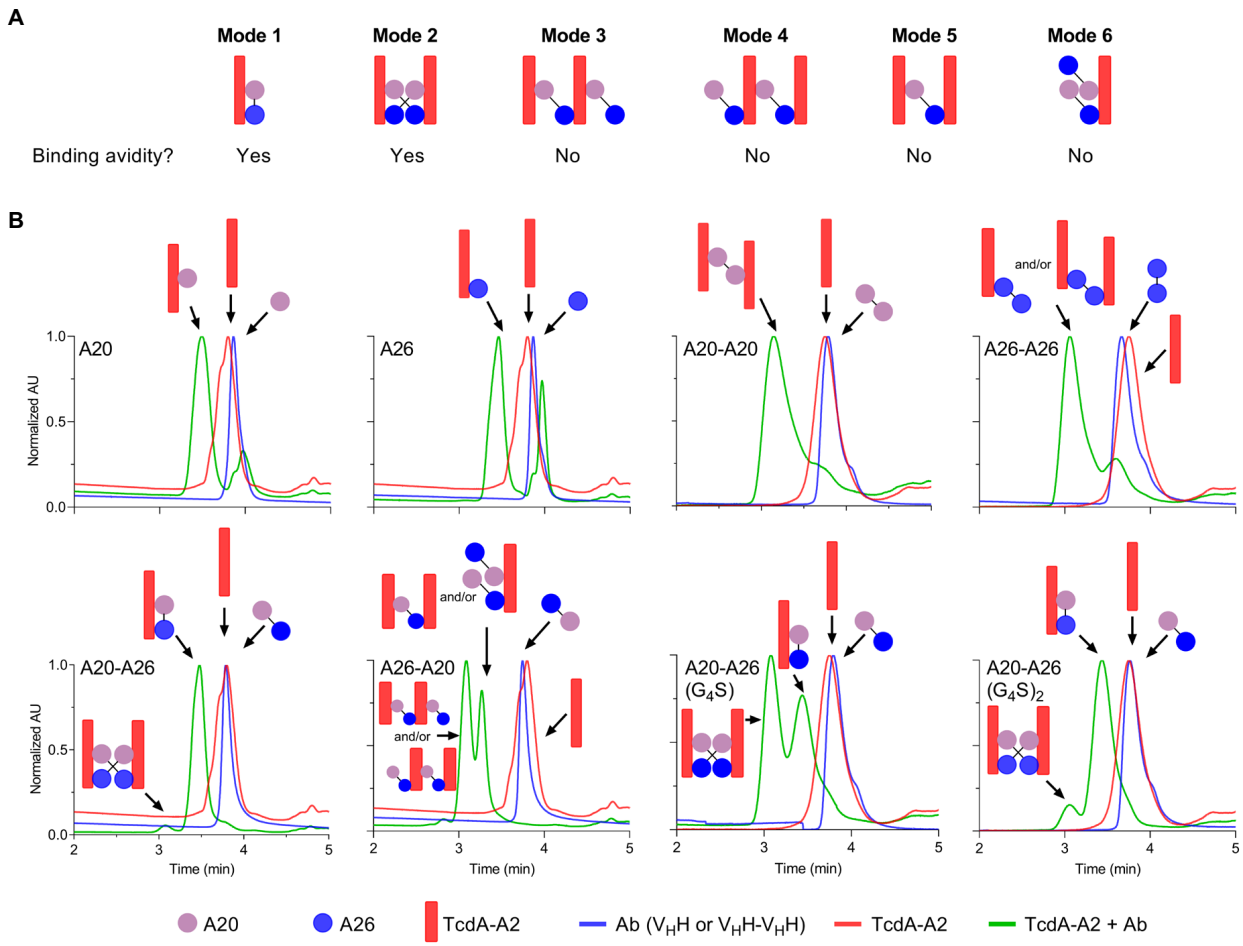
Binding modes and UPLC-SEC chromatograms of TcdA-A2-V_H_H complexes. **(A)** Binding modes for the interaction of TcdA-A2 and heterodimeric V_H_Hs in solution were determined based on molecular mass (*M*_obs_), retention time (*T*_r_) and TcdA neutralization data. **(B)** Chromatograms are shown for TcdA-A2 (red lines), the various antibodies (blue lines) and the 1:1 molar mixture of the two proteins (green lines). The *M*_obs_s of the peak in each chromatogram was estimated from MALS UV. Schematic representations of TcdA-A2, V_H_H or V_H_H-V_H_H and complexes of the two proteins are shown for each peak. AU, absorbance unit.

To gain further insight into the mechanism of action underlying the high neutralization potencies observed with A20-A26 dimers, several monomeric and dimeric V_H_Hs were incubated with TcdA-A2 in solution at 1:1 molar ratios for formation of antibody-TcdA-A2 complexes. The toxin/antibody mixtures were then subjected to SEC-MALS analysis to obtain observed molecular masses (*M*_obs_s) and retention times (*T*_r_s) of free (TcdA-A2 [Ag], antibody [Ab]), and complexed (Ag.Ab) species. *M*_obs_ and *T*_r_ values, complemented with neutralization data, were used to determine the types of binding complexes (“species” and “modes”) formed (Figure 4; Table 3).

**Table 3:** SEC-MALS derived molecular masses for TcdA-A2 + V_H_H complexes.

Sample	*T*_r_ (min)	*M*_r_ (kDa)	*M*_obs_ (kDa)	Species	Relative proportion (%)^a,b^
TcdA-A2	3.748 ± 0.001	32.1	31.3 ± 1.1	Ag	100
A20	3.864 ± 0.001	16.3	17.9 ± 0.1	Ab	100
A26	3.866 ± 0	16.0	18.5 ± 2.1	Ab	100
A20-A20	3.786 ± 0.001	28.7	28.6 ± 2.6	Ab	100
A26-A26	3.665 ± 0.001	29.4	25.4 ± 0.6	Ab	100
A20-A26	3.786 ± 0.001	29.0	29.6 ± 0.6	Ab	100
A26-A20	3.745 ± 0.001	29.0	29.2 ± 0.2	Ab	100
A20-A26 (G_4_S)	3.792 ± 0.001	28.5	26.1 ± 0.5	Ab	100
A20-A26 (G_4_S)_2_	3.767 ± 0.002	28.8	24.1 ± 1.0	Ab	100
TcdA-A2 + A20	3.493 ± 0.001	48.4 [Ag.Ab]	41.1 ± 0	Ag.Ab	79.6
	3.974 ± 0.001	16.3 [Ab]	19.5 ± 0.5	Ab	16.3
	–	–	–	–	4.1
TcdA-A2 + A26	3.470 ± 0	48.1 [Ag.Ab]	41.1 ± 0.2	Ag.Ab	69.2
	3.969 ± 0	16.0 [Ab]	16.4 ± 0.7	Ab	27.0
	–	–	–	–	3.7
TcdA-A2 + A20-A20	3.145 ± 0.004	92.9 [(Ag)_2_.Ab]	77.3 ± 0.9	(Ag)_2_.Ab	92.4
	3.754 ± 0.002	60.8 [Ag.Ab]	65.1 ± 5.9	Ag.Ab	7.5
TcdA-A2 + A26-A26	3.060 ± 0.001	93.6 [(Ag)_2_.Ab]	73.8 ± 0.6	(Ag)_2_.Ab and/or Ag.Ab	76.3
		61.5 [Ag.Ab]			
	3.600 ± 0.003	61.5 [Ag.Ab]	43.5 ± 1.0	Ag.Ab and/or Ag	23.7
		32.1 [Ag]			
TcdA-A2 + A20-A26	3.479 ± 0	61.1 [Ag.Ab]	55.1 ± 0.2	Ag.Ab	91.2
	–	–	–	–	4.7
	–	–	–	–	3.3
TcdA-A2 + A26-A20	3.088 ± 0.001	122.2 [(Ag)_2_.(Ab)_2_]	108.4 ± 0.8	(Ag)_2_.(Ab)_2_	54.6
	3.269 ± 0	93.2 [(Ag)_2_.Ab]	102.6 ± 2.0	(Ag)_2_.Ab and/or Ag.(Ab)_2_	42.7
		90.1 [Ag.(Ab)_2_]			
	–	–	–	–	2.1
TcdA-A2 + A20-A26 (G_4_S)	3.077 ± 0.002	121.4 [(Ag)_2_.(Ab)_2_]	103.3 ± 0.6	(Ag)_2_.(Ab)_2_	46.9
	3.438 ± 0.002	60.6 [(Ag.Ab)]	66.8 ± 2.0	Ag.Ab	53.0
TcdA-A2 + A20-A26 (G_4_S)_2_	3.065 ± 0.003	121.8 [(Ag)_2_.(Ab)_2_]	116.6 ± 0.3	(Ag)_2_.(Ab)_2_	9.2
	3.436 ± 0.003	60.9 [(Ag.Ab)]	56.4 ± 0.6	Ag.Ab	90.8

Ag, TcdA-A2; Ab, V_H_H monomer or dimer depending on sample; *T*_r_, column retention time; *M*_r_, theoretical molecular mass; *M*_obs_, observed molecular mass determined by MALS UV. Values are mean ± SD with from two technical replicates. ^a^Total may not add to 100% in some cases, due to minor peaks not identified in the analysis. Relative proportion is only for the representative data plotted in Figure 4. ^b^Peaks with <5% relative proportion were omitted from species assignment.

As expected, the individual unmixed TcdA-A2 and V_H_H samples treated under the same conditions as the Ag-Ab mixes had *M*_obs_s very close to the theoretical *M*_r_ values, indicating the lack of significant homotypic interactions for each individual protein. The *M*_obs_s for A20:TcdA-A2 and A26:TcdA-A2 complexes were consistent with a 1:1 TcdA-A2-V_H_H complex type (Ag.Ab) and the previous SEC data obtained for these complexes (Murase et al., 2014). The extent of complex formation was higher for A20 (80%) than for A26 (69%), consistent with its higher affinity for TcdA-A2 (Hussack et al., 2011). Homodimeric A20-A20 gave a major complex (92%) of (Ag)_2_.Ab type and a minor complex (8%) of Ag.Ab type. For the A26-A26 homodimer, however, it was not clear from the *M*_obs_s, whether the complex was of Ag.Ab or (Ag)_2_.Ab type for the major SEC species (76%) or of Ag.Ab or Ag type for the minor SEC species (24%) due to intermediary *M*_obs_ values relative to expected *M*_r_s. Nonetheless, all possible complexes point to a lack of binding avidity. Thus, as in the case of A20 *vs* A26 V_H_H, a higher % of complex formation for A20-A20 may be due to a higher intrinsic affinity of A20 for TcdA-A2. Moreover, the lack of binding avidity explains why A26 and A26-A26 have similar potencies, and in the case of A20-A20, it suggests the acquired neutralization capability of A20 upon homodimerization may be due to increased steric hindrance.

A20-A26 formed predominantly an Ag.Ab type complex (91%), and along with its ultra-potent neutralization capability, indicates that both V_H_H domains in A20-A26 simultaneously engage with a single molecule of TcdA-A2 in a biparatopic fashion with 1:1 stoichiometry (Mode 1), as predicted from the original design based on the crystal structure (Figure 4). Conversely, A26-A20 did not appear to form any biparatopic complexes, a result that is also consistent with the crystal structure predictions. It formed two major complexes at 55% and 43% with significantly different *T*_r_s but similar *M*_obs_s, indicative of (Ab)_2_.(Ag)_2_ tetrameric (Modes 2, 3, and 4) and (Ab)_2_.Ag and/or Ab.(Ag)_2_ trimeric complexes (Modes 5 and 6), respectively. Of the three tetrameric possibilities, one can adopt a cross-biparatopic binding arrangement (Mode 2), while the other two arrange in such a manner that excludes binding avidity (Modes 3 and 4, Figure 4). The far weaker neutralization potency of the A26-A20 construct relative to the A20-A26 biparatopic construct, on the one hand, and its similar neutralization capability to monomeric and homodimeric A20/A26 constructs, on the other, indicate that only one V_H_H domain in A26-A20 is able to bind to a single epitope in TcdA-A2 within any one particular molecular complex, thus excluding the possibility of Mode 2 binding. Interestingly, A20-A26 (G_4_S) and A20-A26 (G_4_S)_2_ which had the same V_H_H-V_H_H orientations as A20-A26, formed complexes with *M*_obs_s consistent with Mode 1 and Mode 2 biparatopic Ab.Ag engagement. Molecular models based on the crystal structures indicate that the much shorter linker found in A20-A26 (G_4_S) when compared with A20-A26 would likely prevent the formation of complexes where both of the A20 and A26 V_H_H domains are able to bind to a single molecule of TcdA-A2 simultaneously. This likely explains why only 53% of the complexes detected for A20-A26 (G_4_S) show 1:1 stoichiometry (Ab.Ag; Mode 1), whereas 47% of the complexes show 2:2 stoichiometry ((Ab)_2_.(Ag)_2_; Mode 2) based on the *M*_obs_ and *T*_r_ parameters. In comparison, 91% of the complexes formed by A20-A26 and A20-A26 (G_4_S)_2_, which both have linkers with similar lengths that are expected to allow both V_H_H domains to bind simultaneously to a single molecule of TcdA-A2, show 1:1 stoichiometry (Ab.Ag; Mode 1). Only up to 9% of the complexes have 2:2 stoichiometry ((Ab)_2_.(Ag)_2_; Mode 2). A20-A26 (G_4_S)_3_ was not analyzed by SEC-MALS, because it could not be expressed in sufficient quantities. However, it is expected to possess a similar binding mode to that observed for A20-A26 and A20-A26 (G_4_S)_2_, because the longer linker length would be expected to allow both V_H_H domains to bind to a single molecule of TcdA-A2 simultaneously.

## Discussion

In this work we used the crystal structures of V_H_Hs A20 and A26 in complex with *C. difficle* TcdA that we had previously determined to guide the design of a novel and ultra-potent toxin inhibitor which we have named A20-A26. This fusion protein is comprised of the A20 V_H_H at the N-terminus, followed by a 12 residue (GS)_6_ linker and then the A26 V_H_H at the C-terminus. A20-A26 expressed well in mammalian cells (>100 mg/L), and was monodispersed and free of aggregates. A20-A26 also showed high, avidity-driven apparent binding affinities for immobilized TcdA and TcdA in solution. Most importantly, A20-A26 was extremely potent at neutralizing TcdA in cytotoxicity assays with Vero cells, achieving a ≥ 2,700-fold improvement in potency compared to monomeric A26 and A20 based on *IC*_99_. While this dramatic increase in potency is likely driven by avidity, the steric disruption of receptor binding due to the larger footprint of A20-A26 may also be important.

In contrast, and as predicted from the crystal structures, the reverse orientation of this construct with A26 positioned N-terminal to A20 was much less effective (29,000-fold less potent) at neutralizing TcdA cytotoxicity. The bivalent monoparatopic constructs (A20-A20 and A26-A26), and the anti-TcdA mAb (CDA1 or actoxumab), also show much poorer neutralization activity than A20-A26. To further explore the effects of length and flexibility in the GS-based linkers, we also produced A20-A26 constructs separated by spacer lengths of 5-, 10-, and 15-amino acids. All of these designs were almost as potent as the original version of A20-A26 containing a 12-residue linker, in which the length was suggested by a crude and simplistic model for the linker adopting a fully extended conformation. Combinations of individual V_H_Hs demonstrated poorer efficacy than the top design, pointing to a need for simultaneous engagement of both binding arms by the same antibody molecule to achieve potent cytotoxicity inhibition improvements, an observation reminiscent of analogous multivalent antibody designs evaluated for ricin (Herrera et al., 2015, 2016).

It is important to note that the *IC*_50_ and *IC*_99_ of A20-A26 were essentially the same, 0.16 nM and 0.27 nM, respectively, highlighting the highly cooperative nature of its binding to TcdA. In contrast, for other constructs such as A26, A26-A26, and A26-A20, wide gaps between *IC*_50_ and *IC*_99_ values were observed. In particular, an *IC*_50_ of ~50 nM and an *IC*_99_ of ~7,900 nM, an increase of ~160-fold, was observed for the A26-A20 construct. Based on *IC*_99_ values, A20-A26 showed a 2,700-fold improvement in potency compared to A26 monomer, and a similar improvement with respect to A26-A26. However, it displayed a much higher potency improvement (29,000-fold) relative to A26-A20 due to the much higher *IC*_99_ of A26-A20 (7,866 nM [A26-A20] *vs* 719 nM [A26] *vs* 1,189 nM [A26-A26]). These results suggest that since the A20 moiety binds TcdA more strongly than the A26 moiety of the A26-A20 construct, the A20 moiety may actually sequester the A26 moiety away from the neutralizing A26 epitope, leading to a much higher *IC*_99_ for A26-A20 compared to A26 or A26-A26. While this does not explain why A20, A20-A20, and A26-A20 have similar *IC*_50_s, it does point to the unexpected structural and functional subtleties that arise as a consequence of antibody multimerization, even in the case of very simple multimerization designs such as the linear, flexible linkers used in this study.

SEC-MALS analyses, when considered alongside the neutralization data, reveal that A20-A26 achieves ultra-high TcdA inhibition through a biparatopic interaction with 1:1 stoichiometry (Mode 1; Figure 4A). SEC-MALS also suggests that A20-A26 constructs with 10 [A20-A26 (G_4_S)_2_] and 12 [A20-A26] residue linkers likely have sufficient length and flexibility to allow both V_H_H domains to simultaneously engage with the same molecule of TcdA to form a biparatopic Ab.Ag complex. In comparison, the A20-A26 construct with a shorter five-residue linker [A20-A26 (G_4_S)] does not appear to readily bind a single molecule of TcdA with high affinity, presumably because the shorter linker prevents both V_H_H domains from simultaneously engaging with the same molecule of TcdA. Somewhat surprisingly, this construct is still able to neutralize TcdA with a similar level of potency to A20-A26 and A20-A26 (G_4_S)_2_. SEC-MALS suggests that this is accomplished by two biparatopic V_H_H-V_H_H fusion protein molecules simultaneously engaging with two TcdA molecules in a second, biparatopic (Ab)_2_.(Ag)_2_ interaction (Mode 2), which is presumably nearly as effective a neutralizing mechanism as the intramolecular biparatopic one (Mode 1). Both Mode 1 and Mode 2 binding arrangements involve avidity in solution, i.e., in the toxin neutralization assay setting. This explains why the overall impact of linker length on TcdA neutralization potency for all of these A20-A26 constructs was largely negligible at the TcdA concentrations used in the Vero cell neutralization assays. However, given that Mode 1 biparatopic complex structures are less disturbed by changes in concentrations than Mode 2 biparatopic complex structures, it is likely that A20-A26 would be a more potent inhibitor than A20-A26 (G_4_S) at lower, pathologically relevant, TcdA concentrations. The opposite orientation (A26-A20) and monoparatopic controls (A20-A20, A26-A26) are much less effective at neutralizing TcdA due to a lack of avidity when binding to the toxin, although they did display high apparent binding affinities from bivalent engagement in artificial settings (ELISA, SPR). Based on our previous crystal structures (Ho et al., 2005; Greco et al., 2006; Murase et al., 2014) and the work of others (Pruitt et al., 2010; Chumbler et al., 2016; Chen et al., 2022; Kordus et al., 2022), we hypothesize that the bidentate engagement of A20-A26 with TcdA may lead to ultra-potent toxin inhibition through three possible mechanisms: (i) A20-A26 may block enough of the carbohydrate binding pockets in TcdA to substantially disrupt binding interactions between TcdA and cell-surface carbohydrate receptors; (ii) A20-A26 may inhibit pH-induced TcdA conformational changes that are required for cellular toxicity (Pruitt et al., 2010; Chumbler et al., 2016; Chen et al., 2022); and (iii) we previously deduced the importance of the extreme C-terminus of TcdA playing a role in toxicity (Murase et al., 2014), based on the A26 V_H_H monomer binding at this region of the toxin and neutralizing TcdA relatively effectively (Hussack et al., 2011). It is also instructive to consider studies on anti-ricin antibodies, where Herrera et al. (2016) showed that separate V_H_H domains in bispecific anti-ricin antibody fusions neutralized the toxin through a mechanism that involved a single antibody fusion binding more than a single toxin molecule.

In conclusion, we have validated a structure-guided approach for designing a highly-potent *C. difficle* TcdA inhibitor. In the process we show the importance of V_H_H orientation and geometry in biparatopic constructs, the impact of linker length, and the mode of V_H_H:toxin interactions on achieving ultra-high neutralization potencies.

## Data availability statement

The original contributions presented in the study are included in the article/supplementary material, further inquiries can be directed to the corresponding authors.

## Author contributions

GH and JT conceived experiments, analyzed data, and wrote the manuscript. MR performed protein expression and purification, SDS-PAGE, SEC, ELISA, and neutralization assays, analyzed data, and edited the manuscript. HF performed SPR experiments and analyzed data. TM and LE performed SEC experiments and contributed reagents. JS designed and analyzed SEC-MALS experiments and edited the manuscript. KN conceived experiments, analyzed data, and edited the manuscript. All authors contributed to the article and approved the submitted version.

## Funding

This work was supported by internal funding from the National Research Council Canada, Project Grant TP4 from the Canadian Glycomics Network to KN and JT, and NSERC Discovery Grant 05287 to KN.

## Conflict of interest

The authors declare that the research was conducted in the absence of any commercial or financial relationships that could be construed as a potential conflict of interest.

## Publisher’s note

All claims expressed in this article are solely those of the authors and do not necessarily represent those of their affiliated organizations, or those of the publisher, the editors and the reviewers. Any product that may be evaluated in this article, or claim that may be made by its manufacturer, is not guaranteed or endorsed by the publisher.

## Footnotes

^1^ https://www.cdc.gov/drugresistance/pdf/threats-report/clostridioides-difficile-508.pdf

## Abbreviations

CROP: combined repetitive oligopeptide domain of TcdA or TcdB
ELISA: enzyme-linked immunosorbent assay
PBS: phosphate-buffered saline
SEC-MALS: Size-exclusion chromatography-multiangle light scattering
SDS-PAGE: sodium dodecyl sulfate-polyacrylamide gel electrophoresis
SPR: surface plasmon resonance
TcdA: *Clostridioides difficile* toxin A
TcdB: *Clostridioides difficile* toxin B
V_H_H: camelid heavy-chain antibody variable domain

## References

[ref1] Chen B. Basak S. Chen P. Zhang C. Perry K. Tian S.. (2022). Structure and conformational dynamics of *Clostridioides difficile* toxin A. Life Sci. Alliance 5:e202201383. doi: 10.26508/lsa.202201383, PMID: 35292538 PMC8924006

[ref2] Chen P. Lam K. H. Liu Z. Mindlin F. A. Chen B. Gutierrez C. B.. (2019). Structure of the full-length *Clostridium difficile* toxin B. Nat. Struct. Mol. Biol. 26, 712–719. doi: 10.1038/s41594-019-0268-0, PMID: 31308519 PMC6684407

[ref3] Chumbler N. M. Rutherford S. A. Zhang Z. Farrow M. A. Lisher J. P. Farquhar E.. (2016). Crystal structure of *Clostridium difficile* toxin A. Nat. Microbiol. 1:15002. doi: 10.1038/nmicrobiol.2015.2, PMID: 27571750 PMC4976693

[ref4] Durocher Y. Perret S. Kamen A. (2002). High-level and high-throughput recombinant protein production by transient transfection of suspension-growing human 293-EBNA1 cells. Nucleic Acids Res. 30, 9e–99e. doi: 10.1093/nar/30.2.e9, PMID: 11788735 PMC99848

[ref5] Fu Y. Luo Y. Grinspan A. M. (2021). Epidemiology of community-acquired and recurrent *Clostridioides difficile* infection. Ther. Adv. Gastroenterol. 14:175628482110162. doi: 10.1177/17562848211016248 PMC8141977 34093740

[ref6] Greco A. Ho J. G. Lin S. J. Palcic M. M. Rupnik M. Ng K. K. (2006). Carbohydrate recognition by *Clostridium difficile* toxin A. Nat. Struct. Mol. Biol. 13, 460–461. doi: 10.1038/nsmb1084, PMID: 16622409

[ref7] Henderson M. Bragg A. Fahim G. Shah M. Hermes-DeSantis E. R. (2017). A review of the safety and efficacy of vaccines as prophylaxis for *Clostridium difficile* infections. Vaccines (Basel) 5:25. doi: 10.3390/vaccines5030025 28869502 PMC5620556

[ref8] Herrera C. Klokk T. I. Cole R. Sandvig K. Mantis N. J. (2016). A bispecific antibody promotes aggregation of ricin toxin on cell surfaces and alters dynamics of toxin internalization and trafficking. PLoS One 11:e0156893. doi: 10.1371/journal.pone.0156893, PMID: 27300140 PMC4907443

[ref9] Herrera C. Tremblay J. M. Shoemaker C. B. Mantis N. J. (2015). Mechanisms of ricin toxin neutralization revealed through engineered homodimeric and heterodimeric camelid antibodies. J. Biol. Chem. 290, 27880–27889. doi: 10.1074/jbc.M115.658070, PMID: 26396190 PMC4646030

[ref10] Hmila I. Abdallah R. B. Saerens D. Benlasfar Z. Conrath K. Ayeb M. E.. (2008). VHH, bivalent domains and chimeric heavy chain-only antibodies with high neutralizing efficacy for scorpion toxin AahI. Mol. Immunol. 45, 3847–3856. doi: 10.1016/j.molimm.2008.04.011, PMID: 18614235

[ref11] Hmila I. Saerens D. Ben Abderrazek R. Vincke C. Abidi N. Benlasfar Z.. (2010). A bispecific nanobody to provide full protection against lethal scorpion envenoming. FASEB J. 24, 3479–3489. doi: 10.1096/fj.09-148213, PMID: 20410443

[ref12] Ho J. G. Greco A. Rupnik M. Ng K. K. (2005). Crystal structure of receptor-binding C-terminal repeats from *Clostridium difficile* toxin A. Proc. Natl. Acad. Sci. U. S. A. 102, 18373–18378. doi: 10.1073/pnas.0506391102, PMID: 16344467 PMC1317924

[ref13] Huang N. J. Pishesha N. Mukherjee J. Zhang S. Deshycka R. Sudaryo V.. (2017). Genetically engineered red cells expressing single domain camelid antibodies confer long-term protection against botulinum neurotoxin. Nat. Commun. 8:423. doi: 10.1038/s41467-017-00448-0, PMID: 28871080 PMC5583347

[ref14] Hussack G. Arbabi-Ghahroudi M. van Faassen H. Songer J. G. Ng K. K. MacKenzie R.. (2011). Neutralization of *Clostridium difficile* toxin A with single-domain antibodies targeting the cell receptor binding domain. J. Biol. Chem. 286, 8961–8976. doi: 10.1074/jbc.M110.198754, PMID: 21216961 PMC3058971

[ref15] Hussack G. Ryan S. van Faassen H. Rossotti M. MacKenzie C. R. Tanha J. (2018). Neutralization of *Clostridium difficile* toxin B with V_H_H-Fc fusions targeting the delivery and CROPs domains. PLoS One 13:e0208978. doi: 10.1371/journal.pone.0208978, PMID: 30540857 PMC6291252

[ref16] Hussack G. Tanha J. (2016). An update on antibody-based immunotherapies for *Clostridium difficile* infection. Clin. Exp. Gastroenterol. 9, 209–224. doi: 10.2147/CEG.S84017, PMID: 27536153 PMC4975149

[ref17] Katsamba P. S. Navratilova I. Calderon-Cacia M. Fan L. Thornton K. Zhu M.. (2006). Kinetic analysis of a high-affinity antibody/antigen interaction performed by multiple Biacore users. Anal. Biochem. 352, 208–221. doi: 10.1016/j.ab.2006.01.034, PMID: 16564019

[ref18] Kim D. Y. Kandalaft H. Ding W. Ryan S. van Faassen H. Hirama T.. (2012). Disulfide linkage engineering for improving biophysical properties of human V_H_ domains. Protein Eng. Des. Sel. 25, 581–590. doi: 10.1093/protein/gzs055, PMID: 22942392

[ref19] Kordus S. L. Thomas A. K. Lacy D. B. (2022). *Clostridioides difficile* toxins: mechanisms of action and antitoxin therapeutics. Nat. Rev. Microbiol. 20, 285–298. doi: 10.1038/s41579-021-00660-2, PMID: 34837014 PMC9018519

[ref20] Kyne L. Warny M. Qamar A. Kelly C. P. (2000). Asymptomatic carriage of *Clostridium difficile* and serum levels of IgG antibody against toxin A. N. Engl. J. Med. 342, 390–397. doi: 10.1056/NEJM200002103420604, PMID: 10666429

[ref21] Kyne L. Warny M. Qamar A. Kelly C. P. (2001). Association between antibody response to toxin A and protection against recurrent *Clostridium difficile* diarrhoea. Lancet 357, 189–193. doi: 10.1016/S0140-6736(00)03592-3, PMID: 11213096

[ref22] Lam K. H. Tremblay J. M. Vazquez-Cintron E. Perry K. Ondeck C. Webb R. P.. (2020). Structural insights into rational design of single-domain antibody-based antitoxins against botulinum neurotoxins. Cell Rep. 30, 2526–2539.e6. doi: 10.1016/j.celrep.2020.01.107, PMID: 32101733 PMC7138525

[ref23] Leav B. A. Blair B. Leney M. Knauber M. Reilly C. Lowy I.. (2010). Serum anti-toxin B antibody correlates with protection from recurrent *Clostridium difficile* infection (CDI). Vaccine 28, 965–969. doi: 10.1016/j.vaccine.2009.10.144, PMID: 19941990

[ref24] Mejías M. P. Hiriart Y. Lauché C. Fernández-Brando R. J. Pardo R. Bruballa A.. (2016). Development of camelid single chain antibodies against Shiga toxin type 2 (Stx2) with therapeutic potential against hemolytic uremic syndrome (HUS). Sci. Rep. 6:24913. doi: 10.1038/srep24913, PMID: 27118524 PMC4847011

[ref25] Moayeri M. Leysath C. E. Tremblay J. M. Vrentas C. Crown D. Leppla S. H.. (2015). A heterodimer of a VHH (variable domains of camelid heavy chain-only) antibody that inhibits anthrax toxin cell binding linked to a VHH antibody that blocks oligomer formation is highly protective in an anthrax spore challenge model. J. Biol. Chem. 290, 6584–6595. doi: 10.1074/jbc.M114.627943, PMID: 25564615 PMC4358291

[ref26] Mukherjee J. Tremblay J. M. Leysath C. E. Ofori K. Baldwin K. Feng X.. (2012). A novel strategy for development of recombinant antitoxin therapeutics tested in a mouse botulism model. PLoS One 7:e29941. doi: 10.1371/journal.pone.0029941, PMID: 22238680 PMC3253120

[ref27] Murase T. Eugenio L. Schorr M. Hussack G. Tanha J. Kitova E. N.. (2014). Structural basis for antibody recognition in the receptor-binding domains of toxins A and B from *Clostridium difficile*. J. Biol. Chem. 289, 2331–2343. doi: 10.1074/jbc.M113.505917, PMID: 24311789 PMC3900976

[ref28] Pruitt R. N. Chambers M. G. Ng K. K. Ohi M. D. Lacy D. B. (2010). Structural organization of the functional domains of *Clostridium difficile* toxins A and B. Proc. Natl. Acad. Sci. U. S. A. 107, 13467–13472. doi: 10.1073/pnas.1002199107, PMID: 20624955 PMC2922184

[ref29] Pruitt R. N. Lacy D. B. (2012). Toward a structural understanding of *Clostridium difficile* toxins A and B. Front. Cell. Infect. Microbiol. 2:28. doi: 10.3389/fcimb.2012.00028 22919620 PMC3417631

[ref30] Rossotti M. A. González-Techera A. Guarnaschelli J. Yim L. Camacho X. Fernández M.. (2015). Increasing the potency of neutralizing single-domain antibodies by functionalization with a CD11b/CD18 binding domain. MAbs 7, 820–828. doi: 10.1080/19420862.2015.1068491, PMID: 26192995 PMC4622952

[ref31] Schmidt D. J. Beamer G. Tremblay J. M. Steele J. A. Kim H. B. Wang Y.. (2016). A tetraspecific VHH-based neutralizing antibody modifies disease outcome in three animal models of *Clostridium difficile* infection. Clin. Vaccine Immunol. 23, 774–784. doi: 10.1128/CVI.00730-15, PMID: 27413067 PMC5014919

[ref32] Shogbesan O. Poudel D. R. Victor S. Jehangir A. Fadahunsi O. Shogbesan G.. (2018). A systematic review of the efficacy and safety of fecal microbiota transplant for *Clostridium difficile* infection in immunocompromised patients. Can. J. Gastroenterol. Hepatol. 2018, 1–10. doi: 10.1155/2018/1394379 PMC6139215 30246002

[ref33] Tremblay J. M. Mukherjee J. Leysath C. E. Debatis M. Ofori K. Baldwin K.. (2013). A single VHH-based toxin-neutralizing agent and an effector antibody protect mice against challenge with Shiga toxins 1 and 2. Infect. Immun. 81, 4592–4603. doi: 10.1128/IAI.01033-13, PMID: 24082082 PMC3837998

[ref34] Tremblay J. M. Vazquez-Cintron E. Lam K. H. Mukherjee J. Bedenice D. Ondeck C. A.. (2020). Camelid VHH antibodies that neutralize botulinum neurotoxin serotype E intoxication or protease function. Toxins (Basel) 12:611. doi: 10.3390/toxins12100611, PMID: 32987745 PMC7598594

[ref35] Vance D. J. Tremblay J. M. Mantis N. J. Shoemaker C. B. (2013). Stepwise engineering of heterodimeric single domain camelid V_H_H antibodies that passively protect mice from ricin toxin. J. Biol. Chem. 288, 36538–36547. doi: 10.1074/jbc.M113.519207, PMID: 24202178 PMC3868766

[ref36] Vasilescu I. M. Chifiriuc M. C. Pircalabioru G. G. Filip R. Bolocan A. Lazăr V.. (2021). Gut dysbiosis and *Clostridioides difficile* infection in neonates and adults. Front. Microbiol. 12:651081. doi: 10.3389/fmicb.2021.651081 35126320 PMC8810811

[ref37] Vrentas C. E. Moayeri M. Keefer A. B. Greaney A. J. Tremblay J. O'Mard D.. (2016). A diverse set of single-domain antibodies (VHHs) against the anthrax toxin lethal and edema factors provides a basis for construction of a bispecific agent that protects against anthrax infection. J. Biol. Chem. 291, 21596–21606. doi: 10.1074/jbc.M116.749184, PMID: 27539858 PMC5076830

[ref38] Wade J. Rimbault C. Ali H. Ledsgaard L. Rivera-de-Torre E. Abou Hachem M.. (2022). Generation of multivalent nanobody-based proteins with improved neutralization of long α-neurotoxins from elapid snakes. Bioconjug. Chem. 33, 1494–1504. doi: 10.1021/acs.bioconjchem.2c00220, PMID: 35875886 PMC9389527

[ref39] Wilcox M. H. Gerding D. N. Poxton I. R. Kelly C. Nathan R. Birch T.. (2017). Bezlotoxumab for prevention of recurrent *Clostridium difficile* infection. N. Engl. J. Med. 376, 305–317. doi: 10.1056/NEJMoa1602615, PMID: 28121498

[ref40] Yang Z. Schmidt D. Liu W. Li S. Shi L. Sheng J.. (2014). A novel multivalent, single-domain antibody targeting TcdA and TcdB prevents fulminant *Clostridium difficile* infection in mice. J. Infect. Dis. 210, 964–972. doi: 10.1093/infdis/jiu196, PMID: 24683195 PMC4192054

