# Corrigendum: Structure-guided design of a potent *Clostridioides difficile* toxin A inhibitor

**DOI:** 10.3389/fmicb.2023.1167817

**Published:** 2023-03-30

**Authors:** Greg Hussack, Martin A. Rossotti, Henk van Faassen, Tomohiko Murase, Luiz Eugenio, Joseph D. Schrag, Kenneth K.-S. Ng, Jamshid Tanha

**Affiliations:** ^1^Life Sciences Division, Human Health Therapeutics Research Centre, National Research Council Canada, Ottawa, ON, Canada; ^2^Department of Biological Sciences, University of Calgary, Calgary, AB, Canada; ^3^Life Sciences Division, Human Health Therapeutics Research Centre, National Research Council Canada, Montréal, QC, Canada; ^4^Department of Chemistry and Biochemistry, University of Windsor, Windsor, ON, Canada; ^5^Department of Biochemistry, Microbiology and Immunology, University of Ottawa, Ottawa, ON, Canada

**Keywords:** biparatopic, *Clostridioides difficile*, inhibitor, nanobody, single-domain antibody, toxin, V_H_H

In the published article, there was an error. In the **Title**, the genus name “*Clostridioides*” was misspelled as “*Clostridiodes*.”

This sentence previously stated:

“Structure-guided design of a potent *Clostridiodes difficile* toxin A inhibitor”

The corrected sentence appears below:

“Structure-guided design of a potent *Clostridioides difficile* toxin A inhibitor”

A correction has been made to the **Abstract** section, first sentence. This sentence previously stated:

“Crystal structures of camelid heavy-chain antibody variable domains (V_H_Hs) bound to fragments of the combined repetitive oligopeptides domain of *Clostridiodes difficile* toxin A (TcdA) reveal that the C-terminus of V_H_H A20 was located 30 Å away from the N-terminus of V_H_H A26.”

The corrected sentence appears below:

“Crystal structures of camelid heavy-chain antibody variable domains (V_H_Hs) bound to fragments of the combined repetitive oligopeptides domain of *Clostridioides difficile* toxin A (TcdA) reveal that the C-terminus of V_H_H A20 was located 30 Å away from the N-terminus of V_H_H A26.”

A correction has been made to the **Keywords** section. This keyword previously stated:

“*Clostridiodes difficile*”

The corrected keyword appears below:

“*Clostridioides difficile*”

A correction has been made to the **Introduction** section, first paragraph. This sentence previously stated:

“*Clostridiodes difficile* is a spore-forming Gram-positive bacterium capable of infecting humans and causing symptoms ranging from mild diarrhea to pseudomembranous colitis”

The corrected sentence appears below:

“*Clostridioides difficile* is a spore-forming Gram-positive bacterium capable of infecting humans and causing symptoms ranging from mild diarrhea to pseudomembranous colitis”

A correction has been made to the **Abbreviations** section, page 2 Footnote. This abbreviation previously stated:

“TcdA, *Clostridiodes difficile* toxin A”

The corrected abbreviation appears below:

“TcdA, *Clostridioides difficile* toxin A”

A correction has been made to the **Abbreviations** section, page 2 Footnote. This Abbreviation previously stated:

“TcdB, *Clostridiodes difficile* toxin B”

The corrected abbreviation appears below:

“TcdB, *Clostridioides difficile* toxin B”

A correction has been made to the **Figure 1** legend, first sentence. This sentence previously stated:

“Model of the A20-A26 fusion protein bound to the CROPs domain of *Clostridiodes difficile* TcdA.”

The corrected sentence appears below:

“Model of the A20-A26 fusion protein bound to the CROPs domain of *Clostridioides difficile* TcdA.”

The authors apologize for these errors and state that this does not change the scientific conclusions of the article in any way. The original article has been updated.

